# White coat status is a predictive marker for post-esophageal endoscopic submucosal dissection stricture: a retrospective study

**DOI:** 10.1007/s10388-019-00659-y

**Published:** 2019-03-05

**Authors:** Keitaro Takahashi, Mikihiro Fujiya, Nobuhiro Ueno, Takeshi Saito, Yuya Sugiyama, Yuki Murakami, Takuya Iwama, Takahiro Sasaki, Masami Ijiri, Kazuyuki Tanaka, Aki Sakatani, Katsuyoshi Ando, Yoshiki Nomura, Shin Kashima, Mitsuru Goto, Kentaro Moriichi, Toshikatsu Okumura

**Affiliations:** 1 Division of Gastroenterology and Hematology/Oncology, Department of Medicine, Asahikawa Medical University, 2-1 Midorigaoka-higashi, Asahikawa, Hokkaido 078-8510 Japan; 2 Department of Gastroenterology, Asahikawa City Hospital, Asahikawa, Japan; 3 Department of Gastroenterology, Asahikawa Kosei General Hospital, Asahikawa, Japan

**Keywords:** Esophageal stricture, White coat, Endoscopic submucosal dissection, Prevention, Prediction

## Abstract

**Background:**

Steroid therapy is primarily used to prevent esophageal stricture after endoscopic submucosal dissection (ESD). However, esophageal stricture can still occur after preventive therapy, and the effect of preventive steroid therapy cannot be predicted before stricture formation. This study aimed to clarify the risk factors for esophageal stricture after preventive steroid therapy.

**Methods:**

This was a retrospective study conducted at three institutions. From January 2011 to February 2018, 28 large-sized SENs in 26 patients who had a mucosal defect that involved more than three-quarters of the esophageal circumference were enrolled. We classified white coats on artificial ulcers after esophageal ESD into three groups (thin, moderately thick, thick) based on endoscopic images obtained on postoperative day 7.

**Results:**

The white coat status on the artificial ulcer after ESD was a significant risk factor for post-ESD stricture (*p* < 0.05). The stricture rates in patients with thin, moderately thick and thick white coats were 10.0, 36.4 and 85.7%, respectively. When thin and moderately thick white coats were combined, the stricture rate was 23.8%. The rate of stricture in lesions with thick white coats was significantly higher than that in patients with thin white coats or thin to moderately thick white coats (*p* < 0.05). The multivariate analysis revealed that the white coat status was an independent factor related to esophageal stricture (odds ratio 13.70, 95% confidence interval 1.22–154.0; *p* = 0.034).

**Conclusions:**

The thickness of the white coat is a useful marker for predicting the risk of post-ESD stricture and the effectiveness of preventive steroid therapy.

## Background

Although endoscopic submucosal dissection (ESD) has enabled the en bloc resection of large-sized superficial esophageal neoplasms, esophageal ESD has a possibility of causing esophageal stricture, which results in dysphagia, vomiting and weight loss [1, 2]. The circumferential extension of the mucosal defect, longitudinal length of the mucosal defect and histological depth have been reported to be risk factors for esophageal stricture after ESD [3–5]. The esophageal stricture rate of mucosal defects involving more than three-quarters of the esophageal circumference is reported to be 68–92% [6–10]. Thus, stricture prevention is recommended for such cases under the national guidelines of the Japan Esophageal Society [11]. Local steroid injection or oral steroid has primarily been used to prevent esophageal stricture in clinical studies [1, 12, 13]. However, esophageal stricture can still occur after preventive therapy, and there are no ways to predict the effect of preventive steroid therapy before stricture formation.

We investigated the status of the white coat and blood vessels on artificial ulcers after esophageal ESD, which might be a useful marker of esophageal stricture. In the present study, we reviewed superficial esophageal neoplasms (SENs) in patients who were treated by ESD and analyzed the risk factors for esophageal stricture after preventive steroid therapy.

## Methods

### Study patients

We retrospectively reviewed a total of 163 consecutive lesions in 148 patients with superficial esophageal neoplasms (SENs) who underwent ESD at Asahikawa Medical University Hospital, Asahikawa Kosei General Hospital and Asahikawa City Hospital from January 2011 to February 2018; among these cases, mucosal defects that involved more than three-quarters of the esophageal circumference were observed in 34 SENs in 32 patients. We excluded four SENs without preventive steroid therapies and two SENs without scheduled endoscopic examinations after ESD. Finally, 28 SENs in 26 patients (with a mucosal defect that involved more than three-quarters of the esophageal circumference) were enrolled in the present study. This study was approved by the institutional ethics committee of each hospital.

### ESD procedures

ESD was carried out by experienced endoscopists at each institution. A single-channel upper gastrointestinal endoscope (GIF-Q260J; Olympus Medical Systems, Tokyo, Japan) was used with a high-frequency generator (VIO-300D; ERBE Elektromedizin GmbH, Tübingen, Germany). The mode settings of the high-frequency generator were Endocut I (effect 4, duration 3, interval 3) for mucosal incision and forced coagulation (effect 2, 40 W) for submucosal dissection. A FlushKnife (DK2618JB or DK2620J; Fujifilm, Tokyo, Japan) was used for most procedures. Circumferential markings were made outside the tumor margin after iodine staining. Hyaluronic acid solution (Mucoup; Boston Scientific, Tokyo, Japan) was injected into the submucosal layer to lift the surrounding mucosa. The mucosal incision was completed around the markings. Submucosal dissection was then begun from the proximal side to the distal side, and en bloc resection was performed.

### Post-ESD management for stricture prevention

The administration of steroids (oral or locally injected) was used for stricture prevention. Each institutional endoscopist decided the steroid administration protocol with regard to the comorbidities, such as chronic hepatitis B, in each case and contraindications for the use of oral steroids.

In cases in which oral steroids were used, prednisolone was started on postoperative day 2 or 3 at a dose of 30 mg/day. The dose was gradually tapered in decrements of 5 mg/day every 2 weeks. The administration of steroids was discontinued after 12 weeks.

Local steroid injection was performed using triamcinolone acetonide (Kenacort, Bristol-Myers Squibb, Tokyo, Japan) on postoperative days 0, 4 and 7. At each session, 40 mg of triamcinolone acetonide diluted with normal saline was injected into the residual submucosal layer of the resection bed, using a 25-gauge, 4-mm or 1.5-mm needle (TOP Corporation, Tokyo, Japan).

Follow-up endoscopy was scheduled for approximately 1, 4 and 12 weeks after the treatment. When a patient presented with severe dysphagia, an endoscopic examination was performed. Stricture was defined as the failure of a standard endoscope (for the upper gastrointestinal tract) to pass through the treatment site.

### The white coat and blood vessels of artificial ulcers after esophageal ESD

**Fig. 1:**
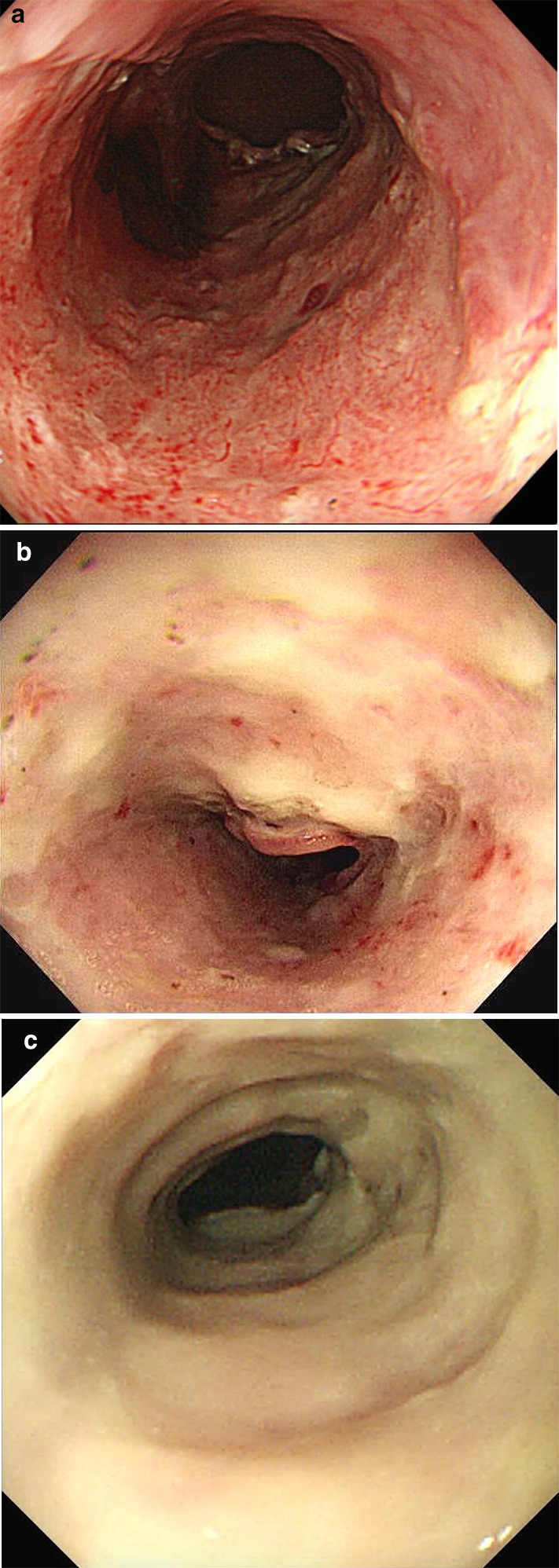
The white coats and blood vessels of the artificial ulcers after esophageal ESD. The groups were defined by visually according to the appearance of the artificial ulcer: in the thin white coat group, blood vessels were clearly visible (**a**); in the moderately thick white coat group, blood vessels that were partially visible (**b**); in the thick white coat group, no blood vessels were visible (**c**)

The thin white coat group included lesions with thin white coats, in which blood vessels were clearly visible on the artificial ulcer (Fig. 1a). The moderately thick white coat group included lesions with moderately thick white coats, in which blood vessels were partially visible on the artificial ulcer (Fig. 1b). The thick white coat group included lesions with thick white coats, in which blood vessels were not visible on the artificial ulcer (Fig. 1c).

An endoscopist with 14 years’ experience performing endoscopy classified white coats into three groups (thin, moderately thick, thick) based on endoscopic images obtained on postoperative day 7. The endoscopist was not involved in the ESD procedure and was blinded to the results of stricture.

### Statistical analyses

All of the statistical analyses were performed using The R Project for Statistical Computing version 3.3.2 software program. The data set of continuous variables was compared using Student’s *t* test, and nominal scale data were compared using Fisher’s exact probability test and Bonferroni correction. Odds ratios (ORs) and 95% confidence intervals (95% CIs) were calculated to evaluate the strength of the influence of each individual variable. The selected variables with *p* values < 0.10 in univariate analysis were included in the multivariate analysis. Multivariate logistic regression analyses were performed to assess the predictive factors of esophageal stricture. *P* values of < 0.05 were considered to indicate statistical significance.

## Results

Table 1 shows the clinicopathological features of the stricture and non-stricture groups. Age, sex, tumor location, circumference, size of tumor, size of resected specimen, depth of invasion, lymphovascular invasion and the ESD procedure time did not differ between the stricture and non-stricture groups. The stricture group included 8 lesions that were treated by local steroid injection and 3 lesions that were treated by oral steroids; while the non-stricture group included 14 lesions treated by local steroid injection and 3 lesions that were treated by oral steroids. There were no significant differences in the preventive therapies of the two groups.

**Table 1:** The clinicopathological features of the stricture and non-stricture groups

	Stricture (*n* = 11)	Non-stricture (*n* = 17)	*P* value
Age, mean ± SD (years)	67.2 ± 10.4	68.2 ± 11.8	0.811
Sex			0.653
Male	8	14	
Female	3	3	
Tumor location			0.121
Cervical esophagus	1	0	
Upper thoracic esophagus	1	2	
Mid-thoracic esophagus	3	11	
Lower thoracic esophagus	6	4	
Circumference			0.076
Circumferential defect	5	2	
3/4 < Circumferential defect	6	15	
Size of tumor, mean ± SD (mm)	43.2 ± 15.6	40.7 ± 17.6	0.707
Size of resected specimen, mean ± SD (mm)	53.0 ± 12.8	52.7 ± 11.5	0.950
Depth of invasion			0.353
T1b	3	2	
T1a	8	15	
Lymphatic invasion	0	2	0.505
Venous invasion	0	1	1
*En bloc* resection	10	17	0.393
ESD procedure time, mean ± SD (min)	151 ± 77	143 ± 57	0.766
Stricture prevention			0.653
Oral steroid administration	3	3	
Local steroid injection	8	14	
White coat			0.007
Thick	6	1	
Moderately thick	4	7	
Thin	1	9	

*SD* Standard deviation

The results of the univariate and multivariate analyses for factors predicting esophageal stricture are summarized in Table 2. The only significant difference was in the status of white coat on the artificial ulcer after esophageal ESD (*p* < 0.05) in the univariate analysis. The multivariate analysis assessing two factors with *p* values < 0.10 showed that the white coat status was an independent factor for predicting post-ESD stricture (odds ratio 13.70, 95% confidence interval 1.22–154.0; *p* = 0.034), whereas the degree of circumferential defect was not.

**Table 2:** Predictors associated with the esophageal stricture

Variables	Univariate analysis		Multivariate analysis	
	OR (95% CI)	*P*	OR (95% CI)	*P*
Age (≥ 65 vs. < 65*) (years)	0.74 (0.11–5.07)	1		
Sex (male vs. female*)	0.58 (0.06–5.43)	0.653		
Tumor location (Ce-Ut vs. Mt-Lt*)	1.64 (0.10–26.35)	1		
Circumference (entire vs. 3/4 < *)	5.80 (0.71–76.95)	0.076	3.13 (0.34–28.7)	0.312
Size of tumor (≥ 40 vs. < 40 mm*)	0.52 (0.08–3.03)	0.460		
Size of resected specimen (≥ 50 vs. < 50 mm*)	0.85 (0.14–5.09)	1		
Depth of invasion (pT1b vs. pT1a*)	2.70 (0.25–38.82)	0.353		
time (≥ 150 vs. < 150 min*)	1.68 (0.29–10.32)	0.700		
Stricture prevention (OS vs. LSI*)	1.71 (0.18–16.12)	0.653		
White coat (Thick vs. Thin-Mod*)	16.86 (1.53–930.07)	0.007	13.70 (1.22–154.00)	0.034

An asterisk was entered in the reference category for each variable to estimate the stricture odds ratio

*OR* Odds ratios, *95% CI* 95% confidence interval, *Ce* cervical esophagus, *Ut* upper thoracic esophagus, *Mt* mid-thoracic esophagus, *Lt* lower thoracic esophagus, *LSI* local steroid injection, *OS* oral steroid, *Thin*-*Mod* thin-moderately thick white coats

The stricture group included one lesion (9.1%) with a thin white coat, four lesions (36.4%) with a moderately thick white coat and six lesions (54.5%) with a thick white coat, while the non-stricture group included nine lesions (52.9%) with a thin white coat, seven lesions (41.2%) with a moderately thick white coat and one lesion (5.9%) with a thick white coat.

The rates of stricture in the lesions with thin, moderately thick and thick white coats were 10.0% (1/10), 36.4% (4/11) and 85.7% (6/7), respectively. The rate of stricture in lesions with thick white coats was significantly higher than that in patients with thin white coats (*p* < 0.05), while the rate of stricture in lesions with thin and moderately thick white coats did not differ to a statistically significant extent.

When lesions with thin and moderately thick white coats (in combination) were compared to those with thick white coats, the rate of stricture in cases involving thin–moderately thick white coats was 23.8% (5/21), while that in cases involving lesions with thick white coats was 85.7% (6/7); the difference was statistically significant (*p* < 0.05).

## Discussion

This is the first report to describe a correlation between the white coat status and esophageal stricture after preventive steroid therapy. The occurrence of the post-ESD strictures tended to increase in proportion to the thickness of the white coat. A thick white coat was associated with a high stricture rate (85.7%). This means that a thick white coat is regarded as a high-risk factor for the development of post-ESD stricture even after steroid therapy. A thick white coat might also indicate the poor efficacy of preventive steroid therapy; thus, more aggressive and effective preventive therapies are required before the stricture forms (i.e., oral steroids in combination with preventive endoscopic balloon dilatation or local steroid injection combined with oral steroids). In contrast, the rates of stricture in thin white coats and thin–moderately thick white coats were 10.0% and 23.8%, respectively. This means that a thin or thin–moderately thick white coat is regarded as a lower risk factor than a thick white coat, suggesting that a thin white coat may indicate a good response to initial preventive therapy, and that follow-up endoscopy without additional treatment may be sufficient.

During the wound-healing process, the infiltration of inflammatory cells, excessive collagen, disorganized fibrosis, granulation, and scar formation might play a role in stricture formation [12, 14–17]. It has been reported that the ulcer basal granulation under the white coat is composed of infiltrative neutrophils and thus the prevention of initial inflammation is important for avoiding esophageal stricture [14, 16]. When the prevention of these inflammatory processes by steroids succeeds at an earlier stage, it seems to result in less amounts of white coats and a lower stricture rate. On the other hand, an insufficient anti-inflammatory effect of steroids seems to result in larger amounts of white coats and a higher stricture rate. Thus, the thickness of the white coat was expected to be a marker of the risk of post-ESD stricture. With regard to blood vessels, angiogenesis was evident after the first postoperative week [2]. The visibility of blood vessels was considered to be dependent on the thickness of the white coat. The blood vessels were not visible under thick white coats, but clearly visible under a thin white coat, indicating that blood vessel visibility was a supportive marker that could be used to evaluate the status of the white coat.

The present study was associated with several limitations. First, this was a retrospective study, while the characteristics of the patients in the stricture and non-stricture groups did not differ to a statistically significant extent. Second, the sample size was relatively small. The entire circumferential defect tended to form the esophageal stricture (71.4%) (*p* = 0.076). A significant difference might have been observed between whole-circumferential defects and < 3/4 circumferential defects if our sample size had been larger. However, the multivariate logistic regression analysis revealed that the white coat status was an independent risk factor for esophageal stricture. Third, differences in the usage and dosage were noted between local steroid injection and oral steroid administration. However, there were no significant differences in the preventive therapies between the stricture and non-stricture groups, and the white coat status was correlated with post-ESD stricture regardless of the preventive therapies administered. Fourth, the reproducibility, including the inter- and intra-observer agreement, for assessing the white coat status, was not examined. Further prospective multi-institutional studies in larger study populations will be needed to confirm the effectiveness of the white coat thickness as a marker.

## Conclusions

The white coat is a useful marker for predicting the risk of esophageal stricture and the effectiveness of preventive steroid therapy. The evaluation of the white coat by endoscopic examination at 1 week after ESD might provide an opportunity to perform additional preventive therapy before stricture formation in high-risk patients.

### Data availability

The datasets supporting the conclusions of this article can be made available upon request.

## Abbreviations

ESD: Endoscopic submucosal dissection
SEN: Superficial esophageal neoplasm
OR: Odds ratios
95% CI: 95% Confidence interval
